# The rise of TIM‐3: A promising immune target in diffuse midline gliomas

**DOI:** 10.1002/ctm2.1536

**Published:** 2024-01-15

**Authors:** Iker Ausejo‐Mauleon, Sara Nuin, Marta M. Alonso

**Affiliations:** ^1^ Health Research Institute of Navarra (IdiSNA) Pamplona Spain; ^2^ Solid Tumor Program, CIMA‐Universidad de Navarra Pamplona Spain; ^3^ Department of Pediatrics Clínica Universidad de Navarra Pamplona Spain

Despite the poor prognostic of paediatric brain tumours, research specifically focused on these tumours has been historically scarce. Consequently, paediatric patients have been treated with therapeutic regimens based on those of adults, which have failed. It is now known that the biogenesis of these tumours is different from adults’ tumours, not to mention the bio‐physiological differences between paediatric and adult patients. Despite the advances in the knowledge of their molecular characteristics, paediatric Central Nervous System (CNS) tumours continue to be the leading cause of cancer death in children aged 0−16 years.^1^ Diffuse midline gliomas, encompassing diffuse intrinsic pontine gliomas (DIPGs), are the most aggressive paediatric brain tumours. These tumours arise from midline structures, including the pons, thalamus, cerebellum and spinal cord, and are inoperable due to their location in vital and intricate brain structures. Their meagre survival has not changed despite the combination of conventional treatments, including radiation, chemotherapy and targeted therapies, emphasizing the urgent need for effective treatments.^2^

Interestingly, a work recently published in *Cancer Cell* by our group has revealed TIM‐3 as a promising new therapeutic target for the treatment of these tumours.^3^ In that work, we observed that TIM‐3 was highly expressed in both tumour cells and immune cells, mainly microglia and macrophages, in samples from DIPG patients. Furthermore, we demonstrated that TIM‐3 blockade in immunocompetent orthotopic models of DIPG prolonged survival, with 50% of long‐term survivors being disease‐free and acquiring immunological memory. Inhibition of TIM‐3 resulted in a significant increase in the number and proliferative status of microglia, NK cells and CD8^+^ T cells, as well as increased levels of IFN‐γ, GrzB and TNF‐α corresponding to activated phenotypes of NK and T cells. Interestingly, a decrease in the regulatory T‐cell population was observed, leading to an increase in the proinflammatory CD8^+^ T cells: Treg ratio. Chemokine studies demonstrated an increase of the chemotactic chemokines CCL5, CCL2 and CXCL10, and the proinflammatory cytokines IL‐1β and IFN‐γ in the tumour microenvironment. Interestingly, only macrophage and microglia depletion resulted in a total loss of efficacy due to the loss of proinflammatory microglia and T‐cell populations, in addition to chemokines and cytokines, indicating a critical role of these myeloid populations in the therapeutic efficacy of TIM‐3 blockade (Figure 1).

**FIGURE 1:**
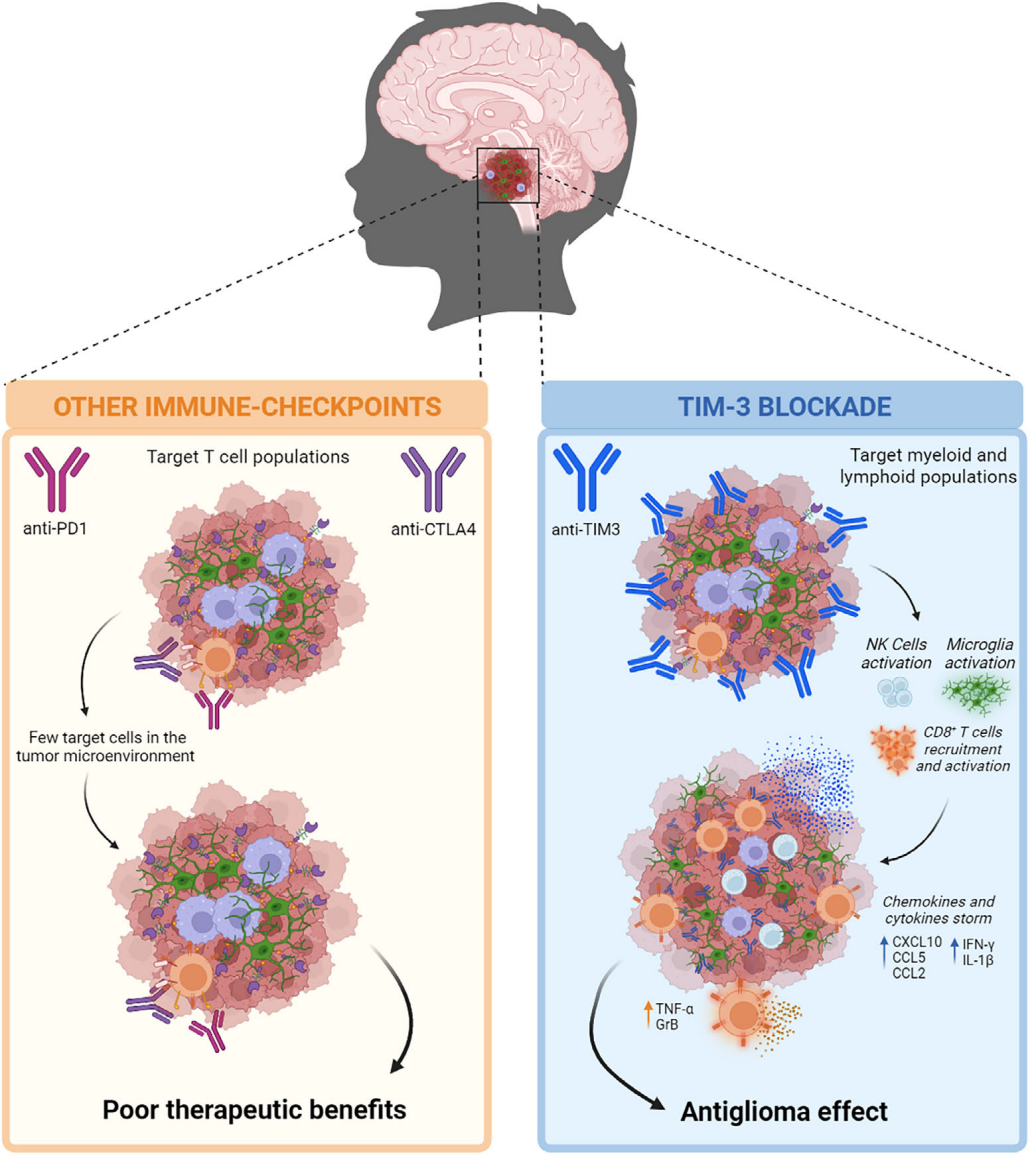
Schematic overview of the mechanism of action of TIM‐3, PD‐1 and CTLA‐4 blockade considering the special immune composition of the tumour microenvironment of DIPGs. Figure created using biorender.com.

In this regard, TIM‐3 blockade offers a completely different approach to the immune checkpoints that have been tested in the clinic without good results, such as anti‐PD1 or anti‐CTLA4 monoclonal antibodies. This was probably due to the poor study of the tumour microenvironment of DIPGs before launching clinical trials with these immunotherapies. The first clinical trial using nivolumab (anti‐PD1) and reRT for the treatment of patients with heavily pretreated DIPG demonstrated good tolerability. Nevertheless, this treatment showed no survival benefit in these patients after PD‐1 blockade.^4^ Despite this lack of efficacy, new phase I and I/II clinical trials with anti‐PD1 (pembrolizumab, NCT02359565; pidilizumab, NCT01952769; and cemiplimab, NCT03690869), anti‐PDL1 (durvalumab, NCT02793466) or anti‐CTLA4+anti‐PD1 (nivolumab+ipilimumab, NCT03130959) have been designed to evaluate the therapeutic efficacy of Immune Checkpoint Blockade (ICB) in DIPG.^5^ However, the therapeutic benefits are far from promising in any of these trials. All these clinical trials have in common that their primary target is T‐cell populations, which is a population that has been observed to be absent in the DIPG tumour microenvironment.^6^ Therefore, considering that the tumour microenvironment of DIPGs is composed mainly of myeloid cells,^7^ it seems much more logical to try a treatment that targets these populations. In our opinion, the future of DIPG immune therapies should be based on two basic principles: to increase the T‐cell infiltrate in the microenvironment and to modulate the myeloid cells present there. We have demonstrated in pre‐clinical models that the therapeutic effect of TIM‐3 is mainly due to its effect on myeloid cells, enhancing the activity of T cells as well,^3^ which makes it a very promising treatment considering the composition of the DIPG tumour microenvironment. Thus, TIM‐3 appears to be a therapy in patients to modulate both the myeloid cells and the T cells that exist in the tumour (Figure 1). Moreover, TIM‐3 blockade is presented as an ideal treatment to be combined with other immunotherapies that increase T‐cell infiltration due to the activation of myeloid cells in the tumour microenvironment and new infiltrating T cells. In this way, virotherapy^8^, ^9^, ^10^ or CAR‐T cells,^11^, ^12^, ^13^ which have already shown good results in clinical trials, may be perfect options to enhance the good therapeutic effect of TIM‐3 blockade as monotherapy.

Most importantly, the lack of other effective therapies for these devastating paediatric brain tumours makes the pre‐clinical results published^3^ especially promising. TIM‐3 blockade appears as a new therapy capable of inducing profound proinflammatory changes in the DIPG myeloid immune populations, leading to the activation of T cells in the tumour microenvironment of these patients. Moreover, these pre‐clinical data offer strong support for initiating a clinical trial with an anti‐TIM‐3 antibody for the treatment of DIPG as monotherapy or even with other already proven treatments, such as oncolytic viruses or CAR‐T cells.

## AUTHOR CONTRIBUTIONS

IAM, SN and MMA contributed equally to this work.

## CONFLICT OF INTEREST STATEMENT

The authors do not have potential conflict of interest to disclose.

## FUNDING INFORMATION

The performed work was supported through a Predoctoral Fellowship from Gobierno de Navarra (VL), Predoctoral Fellowship from Instituto de Salud Carlos III (DdlN), a Postdoctoral Fellowship ChadTough‐Defeat DIPG (MGM), Postdoc Fellowship. Plan de colaboración Internacional (PCI2021‐122084‐2B) Spanish Ministry of Science and Innovation (SL). ChadTough‐Defeat DIPG (MMA), AECC General Projects (PRYGN21937; MMA), Instituto de Salud Carlos III y Fondos Feder (PI19/01896 MMA, PI18/00164 APG “A way to make Europe”); Fundación La Caixa/Caja Navarra (APG and MMA); Fundación El sueño de Vicky; Asociación Pablo Ugarte‐FuerzaJulen, Fundación ADEY, Fundación ACS, (APG and MMA); This project also received funding from the European Research Council (ERC) under the European Union's Horizon 2020 Research and Innovation Programme (817884 ViroPedTher to MMA).

## ETHIC STATEMENT

Not aplicable.
